# The “Biological Weapons” of *Ehrlichia chaffeensis*: Novel Molecules and Mechanisms to Subjugate Host Cells

**DOI:** 10.3389/fcimb.2021.830180

**Published:** 2022-01-14

**Authors:** Yasuko Rikihisa

**Affiliations:** Laboratory of Molecular, Cellular, and Environmental Rickettsiology, Department of Veterinary Biosciences, College of Veterinary Medicine, Infectious Diseases Institute, The Ohio State University, Columbus, OH, United States

**Keywords:** *Ehrlichia chaffeensis*, invasin, ROS, type IV secretion effector, RAB5, autophagy, ferritinophagy, membrane cholesterol

## Abstract

*Ehrlichia chaffeensis* is an obligatory intracellular bacterium that causes human monocytic ehrlichiosis, an emerging, potentially fatal tick-borne infectious disease. The bacterium enters human cells *via* the binding of its unique outer-membrane invasin EtpE to the cognate receptor DNase X on the host-cell plasma membrane; this triggers actin polymerization and filopodia formation at the site of *E. chaffeensis* binding, and blocks activation of phagocyte NADPH oxidase that catalyzes the generation of microbicidal reactive oxygen species. Subsequently, the bacterium replicates by hijacking/dysregulating host-cell functions using Type IV secretion effectors. For example, the *Ehrlichia* translocated factor (Etf)-1 enters mitochondria and inhibits mitochondria-mediated apoptosis of host cells. Etf-1 also induces autophagy mediated by the small GTPase RAB5, the result being the liberation of catabolites for proliferation inside host cells. Moreover, Etf-2 competes with the RAB5 GTPase-activating protein, for binding to RAB5-GTP on the surface of *E. chaffeensis* inclusions, which blocks GTP hydrolysis and consequently prevents the fusion of inclusions with host-cell lysosomes. Etf-3 binds ferritin light chain to induce ferritinophagy to obtain intracellular iron. To enable *E. chaffeensis* to rapidly adapt to the host environment and proliferate, the bacterium must acquire host membrane cholesterol and glycerophospholipids for the purpose of producing large amounts of its own membrane. Future studies on the arsenal of unique *Ehrlichia* molecules and their interplay with host-cell components will undoubtedly advance our understanding of the molecular mechanisms of obligatory intracellular infection and may identify hitherto unrecognized signaling pathways of human hosts. Such data could be exploited for development of treatment and control measures for ehrlichiosis as well as other ailments that potentially could involve the same host-cell signaling pathways that are appropriated by *E. chaffeensis*.

## 1 Introduction

*Ehrlichia chaffeensis* is a tick-borne Gram-negative obligatory intracellular bacterium of the family *Anaplasmataceae* in the order Rickettsiales. Infection causes severe flu-like febrile disease called human monocytic ehrlichiosis (HME), which is often accompanied by hematologic abnormalities and signs similar to those of hepatitis (Dawson et al., 1991; Paddock and Childs, 2003). Tick-borne diseases are on the rise (Biggs et al., 2016; Madison-Antenucci et al., 2020; Alkishe et al., 2021). Discovered in 1986 (Maeda et al., 1987), HME is currently among the most prevalent life-threatening tick-borne zoonoses (Adams et al., 2017). HME diagnosis is challenging, as early signs and symptoms are indistinct or mimic other illnesses. No HME vaccine exists, and the only effective therapy is the broad-spectrum antibiotic doxycycline. However, treatment is often delayed (or even not initiated) owing to misdiagnosis or comorbidity with an unrelated underlying illness or injury, stress, immunosuppression, and/or co-infection with other tick-borne pathogens, which collectively can lead to severe complications or death, with a mortality rate of 1–5% among different populations (Paddock and Childs, 2003). *Ehrlichia* spp. also can negatively impact livestock agroeconomics and working and companion animals, as the various species and strains of *Ehrlichia* can cause severe and potentially fatal diseases in animals (Rikihisa, 1991).

*Ehrlichia chaffeensis* replicates within monocytes and macrophages, which are primary immune cells that recognize pathogen-associated molecular patterns (PAMPs) to unleash potent innate antimicrobial defenses. As a survival strategy, *E. chaffeensis* has lost genes encoding major PAMPs such as lipopolysaccharide, peptidoglycan, flagella, and common pili (Lin and Rikihisa, 2003). It has a single small (1.18 Mbp) circular genome that lacks most genes for amino-acid biosynthesis and intermediary metabolism (Dunning Hotopp et al., 2006); consequently, the bacterium depends on host cells for these molecules. Molecular and cellular research on *E. chaffeensis* has revealed unique mechanisms that mediate its parasitism. Foremost is the *E. chaffeensis* invasin EtpE (entry-triggering protein of *Ehrlichia*), which binds its cognate host-cell surface receptor DNase X, thereby inducing its internalization. This occurs without eliciting host-derived signals that normally would activate the phagocyte NADPH oxidase 2 (NOX2) complex, that catalyzes the production of microbicidal reactive oxygen species (ROS) from molecular oxygen (Mohan Kumar et al., 2013; Teymournejad et al., 2017). Once internalized, the bacterium replicates within a membrane-bound compartment (inclusion); this is also secluded from components of the NOX2 complex (Lin and Rikihisa, 2007).

*Ehrlichia chaffeensis* inclusions rapidly fuse with host-cell early endosomes, thereby acquiring early-endosome markers including the small GTPase RAB5 and its effectors such as early endosome antigen 1, VPS34, and Rabankyrin-5. This facilitates subsequent fusion with other early endosomes that contain transferrin receptor (TfR). Exogenous iron-loaded transferrin (Tf) enters into inclusions through the TfR-Tf endosome recycling pathway (Barnewall et al., 1997). Recent studies revealed that the inclusions have features of the early amphisome, which is the vesicular compartment formed by fusion of early endosomes with early autophagosomes, as ATG5, but not LC3 or ATG14L, was also found in inclusions (Lin et al., 2016). This review primarily focuses on recent findings pertaining to invasin, Type IV secretion system (T4SS) effectors, and host-cell membrane lipids that are acquired by *E. chaffeensis.* Readers are referred to several informative reviews for discussion of other aspects of *E. chaffeensis* (Paddock and Childs, 2003; Rikihisa, 2010; McBride and Walker, 2011; Rikihisa, 2011; Rikihisa, 2015; McClure et al., 2017; Byerly et al., 2021).

## 2 Molecules Unique to *E. chaffeensis* That Facilitate Entry Into Host Cells and Subsequent Intracellular Replication

### 2.1 *Ehrlichia* Entry Is Coupled With Blockade of the Activation of the NOX2 Complex

**Figure 1:**
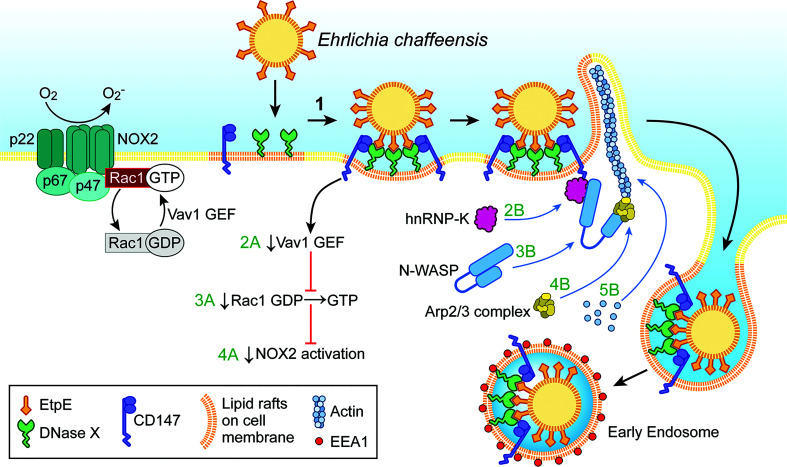
*Ehrlichia* entry is coupled with blockade of the activation of the phagocyte NADPH oxidase (NOX2) complex. Extracellular *E. chaffeensis* uses the C-terminal region of its surface protein EtpE to bind DNase X on the host-cell surface. The consequent lateral redistribution of DNase X within dynamic lipid rafts brings CD147 into association with the EtpE–DNase X complex (1). CD147 relays the signal to downregulate Vav1 GEF (guanine-nucleotide exchange factor; 2A) that prevents Rac1 activation (3A) and consequently prevents activation of the NOX2 complex (4A). CD147 also recruits hnRNP-K to bind N-WASP, leading to activation of N-WASP (conformational change) (2B and 3B). Activated N-WASP binds the Arp2/3 actin-nucleation complex (4B), leading to spatiotemporal actin polymerization and filopodia formation to internalize *E. chaffeensis* into endosomes (5B). The drawing was modified from (Mohan Kumar et al., 2015), copyright 2015 ASM.

As an obligatory intracellular bacterium, *E. chaffeensis* cannot survive without entry into permissive host cells. To enter host monocytes and macrophages, *E. chaffeensis* uses the C-terminus of its unique outer-membrane protein, EtpE-C, to directly bind the host-cell DNase X (DNASE1like1), a cell-surface glycosylphosphatidylinositol-anchored receptor that senses extracellular DNA (Mohan Kumar et al., 2013) (**Figure 1**). Actin polymerization is not required for *E. chaffeensis* binding to host cells but is necessary for entry, and thus entry can be inhibited by cytochalasin D (Mohan Kumar et al., 2015).

EtpE-C-induced actin polymerization is dependent on DNase X as well as activation of the actin nucleation–promoting factor neuronal Wiskott–Aldrich Syndrome protein(N-WASP) (Mohan Kumar et al., 2015). The N-WASP inhibitor wiskostatin or overexpression of the WA domain of N-WASP, which exerts a dominant-negative effect on N-WASP, inhibits actin polymerization and *E. chaffeensis* entry (Mohan Kumar et al., 2015). How does EtpE-C binding to DNase X on the macrophage surface activate cytoplasmic signaling? EtpE-C binding to DNase X on the macrophage surface recruits three factors, namely the type I transmembrane glycoprotein CD147 (basigin/extracellular matrix metalloproteinase inducer), cytoplasmic heterogeneous nuclear ribonucleoprotein K (hnRNP-K), and N-WASP, to facilitate actin polymerization at the site of *E. chaffeensis* binding (Mohan Kumar et al., 2015) (**Figure 1**). CD147 is the key molecule to relay signals started with DNase X membrane receptor to the inside of the cell. Thus, the extracellular bone marrow–derived macrophages from *CD147^flox/flox^*-*Lyz2*-*Cre* mice, in which *Cre* expression (driven by the *Lyz2* promoter) is used for myeloid cell–specific *CD147* knockout, are significantly less susceptible to *E. chaffeensis* infection (Teymournejad and Rikihisa, 2020). hnRNP-K binds N-WASP and activates the Arp2/3 complex to nucleate actin polymerization *in vitro* (Yoo et al., 2006), and the intracellular nanoantibody clone #47 (iAB-47), which binds and confines hnRNP-K to the nucleus (Inoue et al., 2007), potently blocks *E. chaffeensis* entry (Mohan Kumar et al., 2015). Entry requires host-cell energy but not *Ehrlichia* energy, as demonstrated by the fact that latex beads coated with recombinant EtpE-C could bind DNase X and enter phagocytes as well as non-phagocytic cells that are permissive to *E. chaffeensis* infection (Mohan Kumar et al., 2013).

Phagocytes, such as monocytes and neutrophils, produce powerful NADPH oxidase (NOX2 complex), a multicomponent enzyme composed of a membrane-bound heterodimeric cytochrome b558 component (gp91*^phox^* [NOX2] and p22*^phox^*), three cytoplasmic subunits (p67*^phox^*, p47*^phox^*, and p40*^phox^*), and the small GTPase Rac1 or Rac2 (Panday et al., 2015). In resting phagocytes, these NOX2 components are dissociated and hence the enzyme is inactive. Phagocyte-activating agents such as phorbol myristate acetate (PMA), invading pathogens, or an *N*-formyl peptide can induce rapid assembly of all NOX2 components into a holoenzyme that catalyzes the production of superoxide anion $\left({\text{O}}_{2}^{-}\right)$ from molecular oxygen (Debeurme et al., 2010). ${\text{O}}_{2}^{-}$ is secreted extracellularly and into the lumen of phagosomes and serves as starting material for the production of microbicidal reactive oxygen species (ROS), including hydrogen peroxide (H_2_O_2_), oxidized halogens, hydroxyl radicals, and singlet oxygen (Gabig and Babior, 1981). Paradoxically, *E. chaffeensis* isolated from host cells is quite sensitive to ROS, and infectivity decreases rapidly when bacteria are exposed to ROS (Lin and Rikihisa, 2007). In fact, the *E. chaffeensis* genome lacks genes encoding enzymes that facilitate ROS detoxification, free-radical scavenging, repair of ROS-induced damage, and the oxidative stress response (Dunning Hotopp et al., 2006; Lin and Rikihisa, 2007). How, then, does *E. chaffeensis* prevent or the overcome ROS assault by host macrophages? Remarkably, unlike most other bacteria, *E. chaffeensis* does not induce ROS production in human monocytes and rapidly blocks ${\text{O}}_{2}^{-}$ generation induced by PMA (Lin and Rikihisa, 2007). This inhibition is specific to monocytes, as *E. chaffeensis* cannot block ROS production by PMA-stimulated neutrophils, and a host cell-surface protein is required (Lin and Rikihisa, 2007). This surface protein was later revealed to be DNase X, as inhibition of NOX2-complex activation could be initiated by the binding of EtpE-C to DNase X (Teymournejad et al., 2017) (**Figure 1**). Thus, DNase X–mediated entry and ROS blockade are coupled to ensure *E. chaffeensis* survival during entry. However, neutrophils do not express DNase X (Teymournejad et al., 2017), which is likely the primary reason why *E. chaffeensis* neither infects neutrophils nor blocks activation of the NOX2 complex. The binding of *E. chaffeensis* or of recombinant EtpE-C–coated beads to DNase X can trigger activation of N-WASP (Mohan Kumar et al., 2015). However, N-WASP activation is not involved in the blockade of ROS production initiated by *E. chaffeensis* or EtpE-C binding to DNase X (Teymournejad et al., 2017). Rac GTPases act as binary switches for the activation of NOX2 (Seifert et al., 1986; Roberts et al., 1999; Zhao et al., 2003; Bokoch and Zhao, 2006). Two Rac isoforms exist, namely Rac1 and Rac2, and Rac2 is the predominant isoform in human neutrophils, whereas Rac1 predominates in monocytes, the latter accounting for 90% of cellular Rac (Zhao et al., 2003). For Rac activation, GTP-for-GDP exchange is facilitated by a membrane-localized, Rac-specific guanine-nucleotide exchange factor (GEF) (Bokoch et al., 1994), and Rac becomes inactivated upon GTP hydrolysis catalyzed by a GTPase-activating protein specific for Rac (Geiszt et al., 2001). Vav1 is a hemopoiesis-specific Rho/Rac guanine-nucleotide exchange factor that plays a prominent role in adhesion-mediated suppression of ROS generation in phagocytes (Zhao et al., 2003). Engagement of EtpE-C with DNase X triggers CD147-dependent suppression of the PMA-induced activation of Vav1 (Teymournejad and Rikihisa, 2020) (**Figure 1**). Consequently, *E. chaffeensis* and EtpE-C, upon binding DNase X, block Rac1 activation (Teymournejad and Rikihisa, 2020) (**Figure 1**). Actin polymerization led by Rac/wave activation is a well-known mechanism for the entry of several intracellular bacteria including *Listeria*, *Yersinia*, *Salmonella*, and *Chlamydia* into non-phagocytes (Alrutz et al., 2001; Carabeo et al., 2004; Bosse et al., 2007; Humphreys et al., 2013). However, *E. chaffeensis* does not utilize this mode of entry (Mohan Kumar et al., 2015) to colonize phagocytes, as Rac-dependent actin polymerization and entry would activate phagocyte NOX2 as well.

Immunization of mice and dogs with recombinant EtpE-C significantly inhibits *E. chaffeensis* infection *via* intraperitoneal or infected-tick challenge (Mohan Kumar et al., 2013; Budachetri et al., 2020). Thus, EtpE-C could be included in a candidate vaccine to counter tick-transmitted ehrlichiosis.

### 2.2 Functions of *E. chaffeensis* T4SS Effectors

The T4SS can transfer bacterial proteins or nucleoprotein complexes across the membrane of eukaryotic cells (Alvarez-Martinez and Christie, 2009). The T4SS has several ancestral lineages including the archetype *virB/virD* system of *Agrobacterium tumefaciens* and the *dot/icm* system of *Legionella pneumophila*, sometimes referred to as T4aSS and T4bSS, respectively (Alvarez-Martinez and Christie, 2009). All members of the order Rickettsiales, which includes *E. chaffeensis*, have T4aSS (Gillespie et al., 2010). T4SS functions through its effectors/substrates. To date, three T4SS effectors have been experimentally demonstrated, namely *Ehrlichia* translocated factor (Etf)-1, -2, and -3 (Liu et al., 2012; Lin et al., 2016; Yan et al., 2018; Yan et al., 2021).

Etf-1, -2, and -3 directly bind the *E. chaffeensis* T4SS coupling ATPase VirD4 and are then transferred from the bacterium into the host-cell cytoplasm by crossing three membranes (inner and outer *Ehrlichia* membranes, and inclusion membrane) (Liu et al., 2012; Lin et al., 2016; Yan et al., 2018; Yan et al., 2021). Each Etf is required for *E. chaffeensis* infection, as downregulation of any *Etf* gene by electroporation of *E. chaffeensis* with an individual *Etf*-specific antisense peptide nucleic acid significantly reduces the expression of the corresponding mRNA and hence the bacteria’s ability to infect host cells (Sharma et al., 2017; Yan et al., 2018; Yan et al., 2021). This type of inhibition could be trans-complemented by ectopic expression of the corresponding GFP-coupled Etf in host cells, underscoring the critical roles of the three T4SS effectors in *E. chaffeensis* replication (Sharma et al., 2017; Yan et al., 2018; Yan et al., 2021). Characteristics of the three T4SS effectors of *E. chaffeensis* is listed in **Table 1**.

**Table 1:** Characteristics of Type IV secretion effectors from *E. chaffeensis*.

Effector	Amino acid residues	C-terminal residues (basic residues underlined)	Protein motifs	Subcellular localization/functions
Etf-1	380	KHFSNPGKVHAR	Near N-terminal mitochondria localization signal	Mitochondria, bacterial inclusions/ Inhibits mitochondria-mediated apoptosisUpregulates MnSOD Binds Beclin 1 and induces autophagy
Etf-2	264	HARQACGRFFRR	An Arg finger and a Gln finger of Tre2-Bub2-Cdc16 domain	Early endosomes and bacterial inclusions/ Binds RAB5-GTP and blocks RABGAP5 engagement with RAB5-GTP
Etf-3	621	RLSEIFSALTRTIAR	*	Ferritinophagolysosomes/ Binds ferritin light chain and induces ferritinophagy

* Research concerning this motif is ongoing.

#### 2.2.1 Etf-1 Inhibits Host-Cell Apoptosis 

**Figure 2:**
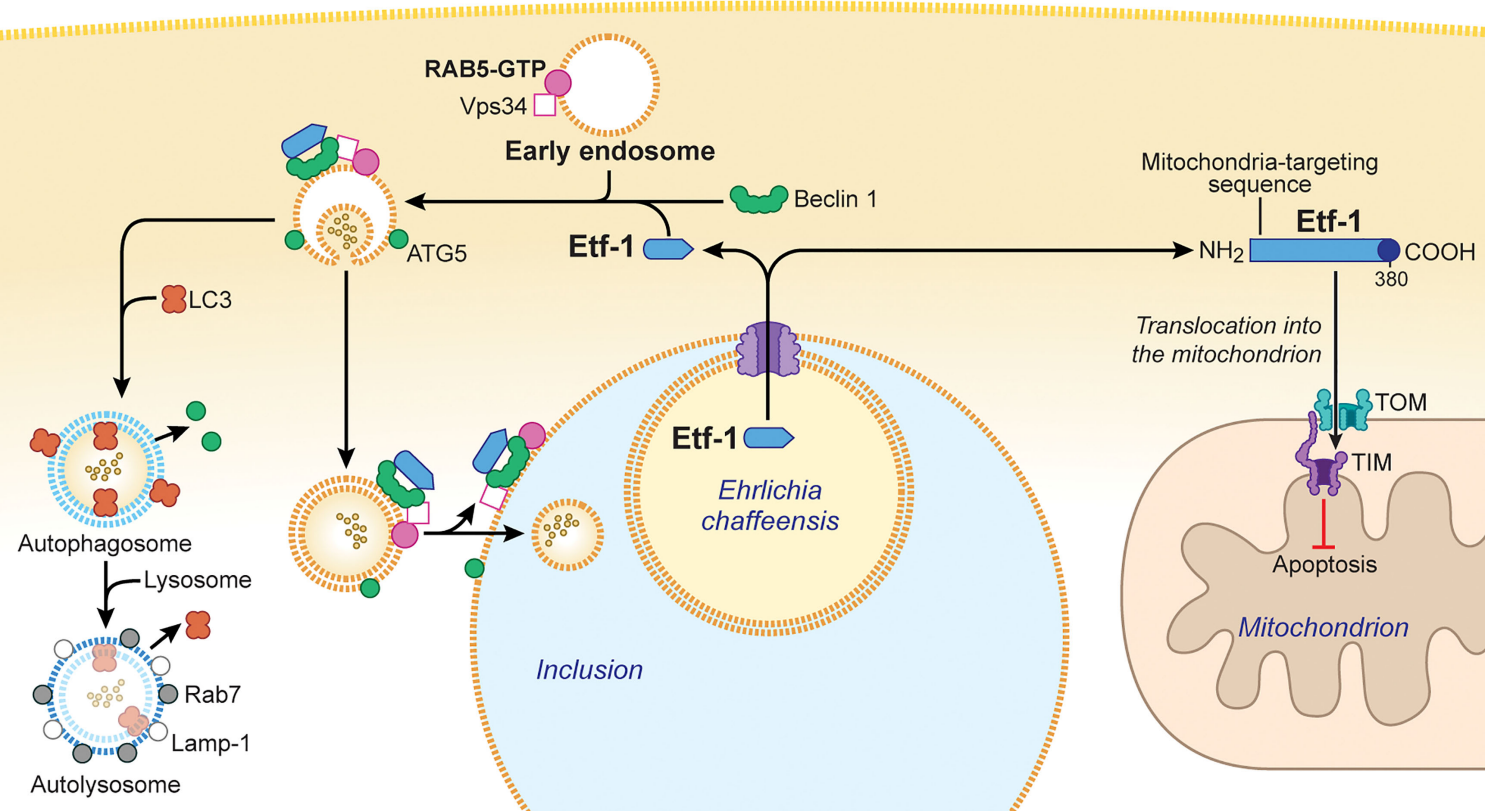
*Ehrlichia chaffeensis* Etf-1 localizes to mitochondria to block apoptosis of host cells. Alternatively, Etf-1 binds to the Belin 1–VPS34–RAB5-GTP complex and induces RAB5-regulated autophagy. (Right) Etf-1 is depicted in blue with the putative T4SS signal depicted in dark blue. Etf-1 has a mitochondria-targeting presequence and localizes to mitochondria. Mitochondria-localized Etf-1 blocks apoptosis of eukaryotic host cells by preventing loss of mitochondrial membrane potential. TOM: transporter outer membrane complex; TIM: transporter inner membrane complex. (Left) Etf-1 binds the Beclin 1–VPS34–RAB5-GTP complex and induces RAB5-regualted autophagy. Etf-1 autophagosomes are recruited to *E. chaffeensis* inclusions and deliver captured host cytoplasmic contents. If not recruited to inclusions, Etf-1 autophagosomes mature to autolysosomes, in which captured substrates are degraded and catabolites are released to the cytoplasmic to promote bacterial proliferation. The drawing was modified from (Rikihisa, 2019), copyright 2017 Taylor & Francis, and from (Rikihisa, 2017), copyright 2017 Springer.

*E. chaffeensis* inhibits host-cell apoptosis to maximize bacterial proliferation inside host cells (Liu et al., 2011; Liu et al., 2012). Etf-1 is highly upregulated during early exponential growth of *E. chaffeensis* in human monocytes (Liu et al., 2012). In Etf-1–transfected mammalian cells, Etf-1 was found to localize to mitochondria and inhibit apoptosis induced by the treatment with etoposide (Liu et al., 2012) (**Figure 2**). Moreover, in similar experiments with yeast, Etf-1 also localized to mitochondria and inhibited apoptosis induced by heterologous expression of human Bax (Liu et al., 2012). The N-terminal 24 amino-acid residues of Etf-1, especially residue K23, play a critical role in mitochondrial targeting of Etf-1, as deletion mutation of this residue significantly decreased Etf-1 localization to mitochondria (Zhang et al., 2021). The mitochondrial matrix protein manganese superoxide dismutase (MnSOD) maintains a basal level of ROS in cells by scavenging ${\text{O}}_{2}^{-}$ and is essential for maintaining aerobic life (Holley et al., 2012). The MnSOD level was found to increase in *E. chaffeensis*–infected cells or Etf-1–transfected cells, and the amount of ROS in infected or Etf-1-transfected cells was significantly lower than that in uninfected or control plasmid–transfected cells (Liu et al., 2012; Yan et al., 2021). These data suggest that, by upregulating mitochondrial MnSOD, Etf-1 serves as an antioxidant to prevent ROS-induced cellular damage and apoptosis to allow intracellular infection (Liu et al., 2012).

To verify the functions of intracellular Etf-1 and investigate the possibility that Etf-1 could be used as a therapeutic target, Etf-1−specific nanobodies were developed by immunizing a llama (Zhang et al., 2021). One particular nanobody could form a stable complex with Etf-1 and thereby block the mitochondrial localization of Etf-1 (Zhang et al., 2021). Intracellular expression of this anti−Etf-1 nanobody inhibited three activities of Etf-1 and *E. chaffeensis*: upregulation of mitochondrial MnSOD, reduction of intracellular ROS, and inhibition of apoptosis (Zhang et al., 2021). Conjugation of this nanobody to cyclized cell-permeable peptide 12 facilitated effective entrance into mammalian cells, where it abrogated the blockade of apoptosis caused by *E. chaffeensis* and inhibited infection by *E. chaffeensis* in cultured cells and in a mouse model of severe combined immunodeficiency (Zhang et al., 2021). Thus, in principle, intracellular nanobodies that interfere with T4SS effector functions could be developed as research tools as well as therapeutic agents.

#### 2.2.2 Etf-1 Induces RAB5-Regulated Autophagy

Autophagy is the process by which eukaryotic cells routinely degrade cellular components to ensure homeostasis and is considered a part of the innate immune response that clears a variety of intracellular pathogens (Levine et al., 2011; Deretic, 2012). However, intracellular replication of *E. chaffeensis* is enhanced by the autophagy inducer rapamycin and inhibited by the autophagy inhibitor 3-methyl adenine (Lin et al., 2016). Use of Spautin-1 (a cell-permeable inhibitor of the autophagy regulator Beclin 1), Beclin 1 small interfering RNA, or mouse bone marrow–derived macrophages from *atg5^flox/flox^*-*Lyz2*-*Cre* mice (in which *Lyz2* promoter–driven *Cre* expression is used for myeloid cell–specific *Atg5* knockout) demonstrated that autophagy not only enhances ehrlichial infection but also is required for *E. chaffeensis* replication (Lin et al., 2016). In fact, *E. chaffeensis* induces a unique type of cellular autophagy to recycle host-cell catabolites for use during its replication (Lin et al., 2016). *E. chaffeensis–*induced autophagy is independent of the general cellular ubiquitination pathways as well as the canonical autophagy pathway involving MTOR, ULK1, and AMPK (Lin et al., 2016). Etf-1 binds Beclin 1 and VPS34 and activates the class III PtdIns3K (phosphatidylinositol 3-kinase) complex, which is an essential component and master regulator of autophagy initiation (**Figure 2**), but this Etf-1 complex does not recruit the endoplasmic reticulum resident ATG14L, unlike Ats-1 of *Anaplasma phagocytophilum*, that also binds Beclin 1 and VPS34 and induces autophagy (Niu et al., 2010). Rather, the Etf-1-Beclin 1 complex recruits RAB5-GTP (Lin et al., 2016) (**Figure 2**). This type of autophagy is referred to as “RAB5-regulated autophagy” (Ravikumar et al., 2008), as constitutively active RAB5 induces autophagy by binding to the RAB5 effector VPS34, which binds Beclin 1 and hence the class III PtdIns3K complex. Expansion of a polyglutamine tract within the Huntingtin protein due to the mutation causes its accumulation and aggregation in the cytoplasm, leading to the neurodegenerative genetic disorder Huntington’s disease (Raspe et al., 2009). The mutant Huntingtin protein, is poorly degraded in proteasomes but can be degraded *via* RAB5-regulated autophagy (Ravikumar et al., 2008). Etf-1–induced RAB5-regulated autophagy was found to clear an aggregation-prone mutant Huntingtin protein in a class III PtdIns3K–dependent manner (Lin et al., 2016).

During the exponential growth stage of *E. chaffeensis*, the concentrations of free/cytoplasmic l-glutamine and l-glutamate in infected human monocytes increase substantially, making them available for ehrlichial growth (Lin et al., 2016). Indeed, host cell–preincorporated radioactive l-glutamine could be readily taken up by *E. chaffeensis* in an autophagy-dependent manner, and the human cytoplasmic autophagy cargo protein GAPDH could be delivered into *E. chaffeensis* inclusions as well (Lin et al., 2016). In addition to several early-endosome markers, Etf-1 and the early autophagosome marker ATG5 (but not LC3) are present on the membrane of *E. chaffeensis* inclusions (Barnewall et al., 1997; Mott et al., 1999; Lin et al., 2016) (**Figure 2**), and thus the inclusions can be considered as large amphisomes formed by fusion of early endosomes and early autophagosomes. Etf-1–induced autophagy releases host-cell small-molecule catabolites into the host cytoplasm to provide nutrients (e.g., amino acids) to *E. chaffeensis*. Furthermore, Etf-1–induced autophagy creates a host cytoplasmic space for *E. chaffeensis* to grow without lysing host cells.

How are the two competing functions of Etf-1 distributed within *E. chaffeensis*–infected cells? The translocase of the outer membrane of mitochondria (TOM) complex is the main pore for the import of nuclear-encoded proteins into mitochondria, and mitochondrial membrane potential is required for import (Pfanner and Truscott, 2002). The majority of Etf-1 targets mitochondria during the early stage of infection when mitochondrial membrane potential is maximal. As infection progresses, Etf-1 is diverted to autophagosomes as mitochondria begin to lose membrane potential (Wurm et al., 2011). This suggests that host-cell physiologic conditions during infection influence the distribution of Etf-1 between mitochondria and autophagosomes, consequently affecting *E. chaffeensis* growth.

Although Etf-1 interacts with RAB5-GTP *via* Beclin 1 and localizes to *E. chaffeensis* inclusions, inhibition of lysosome fusion with inclusions by keeping RAB5 on inclusions, requires another T4SS effector, Etf-2, because Etf-1-GFP vesicles mature to autolysosomes (Lin et al., 2016).

#### 2.2.3 Etf-2 Prevents Lysosomal Fusion of *E. chaffeensis* Inclusions

**Figure 3:**
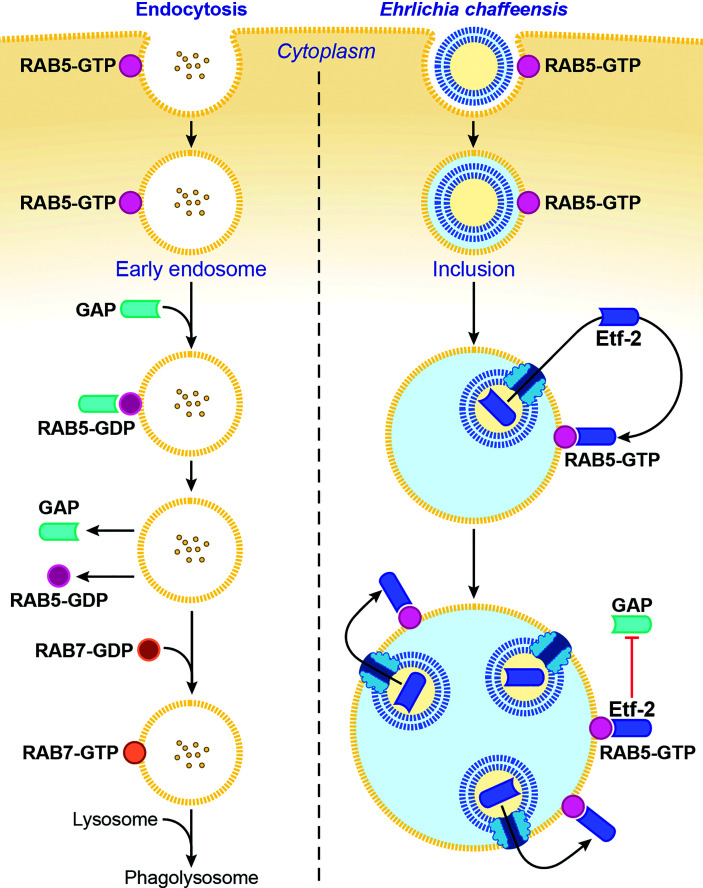
*Ehrlichia chaffeensis* Etf-2 binds RAB5-GTP and blocks RABGAP5 from acting on RAB5. RAB5-GTP hydrolysis by the RAB5-specific GAP is required for endosome maturation and lysosomal fusion (left). Etf-2 is responsible for blocking lysosomal fusion with *E. chaffeensis* inclusions by localizing to *E. chaffeensis* inclusions *via* binding to RAB5-GTP and competitively blocking RABGAP5 from acting on RAB5 on the inclusion surface (right). The drawing is from (Yan et al., 2018), copyright 2018 PNAS.

*Ehrlichia chaffeensis* sequesters the regulator of endosomal traffic, RAB5, on its membrane-bound inclusions to avoid being routed to host-cell phagolysosomes (Barnewall et al., 1997; Mott et al., 1999). How is RAB5 sequestered on the ehrlichial inclusion membrane? The answer is *via* its association with Etf-2. Etf-2 directly binds RAB5-GTP on the membrane of early endosomes and of *E. chaffeensis*–containing inclusions (Yan et al., 2018) (**Figure 3**). A yeast two-hybrid assay and a microscale thermophoresis assay revealed that Etf-2 binds tightly to RAB5-GTP but not RAB5-GDP (Yan et al., 2018). This is because Etf-2 contains two conserved motifs of RAB GAP Tre2-Bub2-Cdc16 domain, namely an Arg finger and a Gln finger, although it lacks RAB5-specific GAP activity (Yan et al., 2018). Thus, Etf-2 binding to RAB5-GTP blocks RAB5-GTP engagement with RABGAP5 (**Figure 3**), and consequently RAB5-GTP hydrolysis is delayed on *E. chaffeensis* inclusions (Yan et al., 2018).

#### 2.2.4 Etf-3 Induces Ferritinophagy

**Figure 4:**
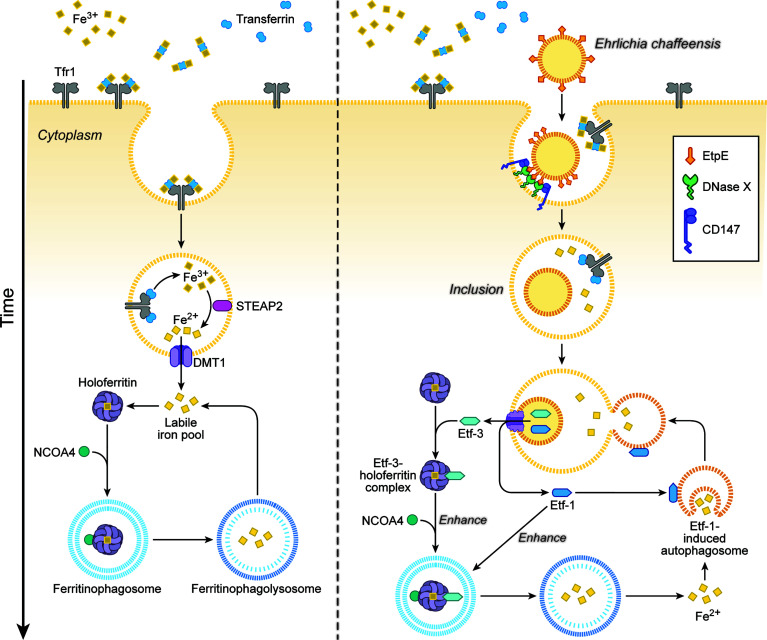
*Ehrlichia chaffeensis* Etf-3 binds ferritin light chain to induce ferritinophagy to increase the labile-iron pool for acquisition of iron by *E. chaffeensis*. Iron homeostasis is tightly regulated in host cells to maintain the labile cellular iron pool (left). Etf-3 directly binds ferritin *via* ferritin light chain and induces ferritinophagy to increase the labile cellular iron pool, thereby providing Fe^2+^ for *E. chaffeensis* proliferation. Etf-1–induced autophagy synergizes with Etf-3 to deliver extra Fe^2+^ to *Ehrlichia* inclusions (right). Tf: transferrin, which binds Fe^3+^ and transports it into cells. TfR: transferrin receptor, which binds and delivers iron-saturated transferrin *via* endocytosis. STEAP2: Six-transmembrane epithelial antigen of prostate-2, a metalloreductase that reduces Fe^3+^ to Fe^2+^. DMT1: Divalent metal transporter 1 that transports Fe^2+^ from endosomes to the cytoplasm. NCOA4: Nuclear receptor coactivator 4, a cargo receptor that mediates ferritinophagy. The drawing is from (Yan et al., 2021), copyright 2021 PNAS.

*Ehrlichia* is an obligate aerobe that requires the electron transport chain, thus iron, because its glycolytic pathway is incomplete and it lacks ATP-ADP translocase, unlike *Rickettsia* and *Chlamydia* (Dunning Hotopp et al., 2006). *Ehrlichia chaffeensis* lacks the siderophore biosynthesis pathway and Fe^3+^ uptake regulator (Dunning Hotopp et al., 2006). Instead, *Ehrlichia* acquires iron from the host-cell labile cellular iron pool, and pretreating human monocytes with deferoxamine, a membrane-permeable chelator of this iron pool, blocks *E. chaffeensis* infection (Barnewall and Rikihisa, 1994). *Ehrlichia* enhances host-cell iron uptake *via* upregulating TfR mRNA (Barnewall et al., 1999) and acquires iron from holoTf, as *E. chaffeensis* endosomes intersect with TfR-recycling endosomes and are slightly acidic—enough to release iron from holoTf (Barnewall et al., 1997). In fact, treatment of macrophages with interferon-γ downregulates TfR mRNA and almost completely inhibits *Ehrlichia* infection, and addition of holoTf abrogates this inhibition (Barnewall and Rikihisa, 1994). However, TfR mRNA levels return to basal level after 24 h post-infection, when bacterial exponential growth begins (Barnewall et al., 1999); at that time, treatment with interferon-γ can no longer inhibit infection (Barnewall and Rikihisa, 1994). How, then, does exponentially growing *Ehrlichia* acquire iron from host cells? The answer is Etf-3, which binds directly and tightly to ferritin (the primary eukaryotic cytoplasmic iron storage protein) and thereby induces ferritinophagy, a selective form of autophagy by recruiting NCOA4 (nuclear receptor coactivator 4), a cargo receptor that mediates ferritinophagy, and LC3, an autophagosome biogenesis protein (Yan et al., 2021) (**Figure 4**). Etf-3–induced ferritinophagy causes ferritin degradation and significantly increases the labile cellular iron pool, which can feed *E. chaffeensis* (**Figure 4**). Indeed, an increase in cellular ferritin by adding ferric ammonium citrate to the culture medium, or overexpression of Etf-3 or NCOA4, enhances *E. chaffeensis* proliferation, whereas knockdown of Etf-3 in *Ehrlichia via* transfection with a plasmid encoding an Etf-3 antisense peptide nucleic acid inhibits *Ehrlichia* proliferation (Yan et al., 2021).

Excessive ferritinophagy induces the generation of toxic ROS, which could presumably kill both *Ehrlichia* and host cells. During *Ehrlichia* proliferation, however, there is concomitant upregulation of *Ehrlichia* Fe-superoxide dismutase, the gene that is co-regulated with the *Ehrlichia* T4SS operon, and increase in mitochondrial MnSOD in response to the co-secreted Etf-1 (Yan et al., 2021). Consequently, despite enhanced ferritinophagy, cellular ROS levels are reduced in *Ehrlichia-*infected cells compared with uninfected cells (Yan et al., 2021). Thus, *Ehrlichia* robs host-cell iron sequestered in ferritin without killing the host cell.

## 3 Hijacking Host Membrane Lipids (Cholesterol and Glycerophospholipid) by *E. chaffeensis*

**Figure 5:**
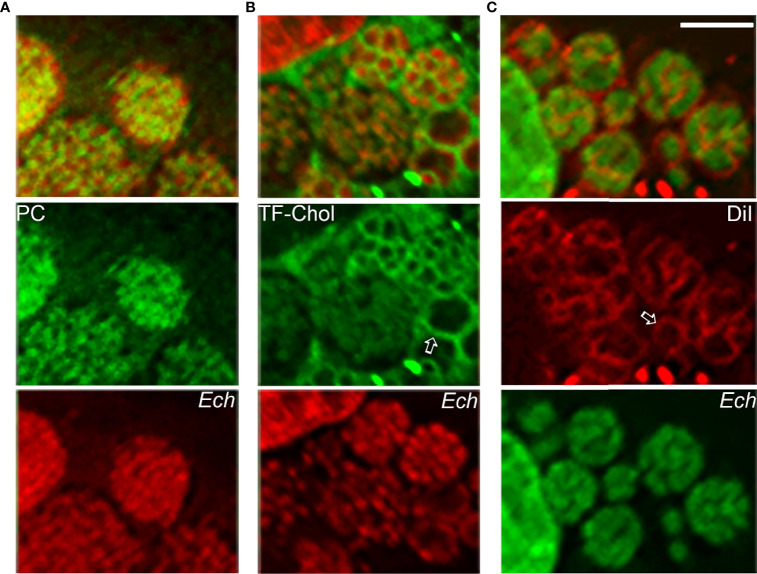
Host-cell membrane lipids (cholesterol and glycerophospholipids) are trafficked to the membrane of *E. chaffeensis* and its inclusions. NBD-phosphatidylcholine **(A)**, TopFluor-cholesterol **(B)**, or DiI **(C)** is added to *E. chaffeensis*–infected RF/6A cells to monitor the intracellular distribution of the fluorescent lipids. DNAs of bacteria and host are stained with Hoechst 33342 (pseudocolored green and red, respectively). Note labeling of densely packed intraluminal *E. chaffeensis* (*Ech*, small cocci stained with Hoechst 33342) with the three fluorescent lipids **(A–C)** and labeling of the bacterial inclusion membrane (open arrows) and intraluminal membranes with TopFluor-cholesterol **(B)** and DiI **(C)**. Bar: 3 µm. The drawing was modified from (Lin et al., 2020), copyright 2020 PNAS.

The *E. chaffeensis* cell membrane is cholesterol-rich (Lin and Rikihisa, 2003), but the bacterium cannot synthesize cholesterol and partially lacks genes for glycerophospholipid biosynthesis (Lin et al., 2020). As small Gram-negative bacteria, *Ehrlichia* spp. require abundant membrane lipids for rapid intracellular proliferation. Thus, *E. chaffeensis* must acquire these membrane lipids from host cells. Furthermore, by incorporating eukaryotic lipids such as phosphatidylcholine and cholesterol, *E. chaffeensis* mimics the eukaryotic plasma membrane and, by doing so, adapts to the cellular environment of the host. Indeed, exogenous 7-nitrobenz-2-oxa-1,3-diazol-4-yl (NBD)-phosphatidylcholine, Bodipy-phosphatidylethanolamine, and Bodipy (TopFluor)-cholesterol are rapidly trafficked to ehrlichia inclusions in infected cells (**Figure 5**). DiI (3,3’-dioctadecylindocarbocyanine)-prelabeled host-cell membranes are unidirectionally trafficked to *Ehrlichia* inclusions and the bacterial membrane (**Figure 5**), but DiI-prelabeled *Ehrlichia* membranes are not reversibly trafficked to host-cell membranes (Lin et al., 2020). The trafficking of host-cell membranes to *Ehrlichia* inclusions is dependent on both the host endocytic and autophagic pathways as well as bacterial protein synthesis, as the respective inhibitors block the trafficking of DiI-labeled host membranes to *Ehrlichia* as well as infection (Lin et al., 2020). Cryosections of infected cells show numerous membranous vesicles inside *Ehrlichia* inclusions as well as multivesicular bodies docked on the inclusion surface, both of which can be labeled by GFP-tagged 2×FYVE protein that binds to phosphatidylinositol 3-phosphate, a product of PtdIns3K activity (Lin et al., 2020). Focused ion-beam scanning electron microscopy of infected cells has validated the existence of numerous membranous structures inside bacterial inclusions (Lin et al., 2020). These results support the notion that *Ehrlichia* inclusions are amphisomes formed through fusion of early endosomes, multivesicular bodies, and early autophagosomes induced by Etf-1, and they provide the host-cell membrane glycerophospholipids and cholesterol necessary for bacterial proliferation.

## 4 Future Directions

*Ehrlichia* species propagate *via* perpetual transmission between ticks and mammalian hosts and can proliferate in each of these two distinct environments. Owing to multiple technical limitations, little is known about the bacterial components that enable *Ehrlichia* to thrive throughout this lifecycle. Recently, Himer1 transposon mutagenesis was successfully applied to *E. chaffeensis* as well as other *Ehrlichia* species, and functional knockout mutants have been cloned (Cheng et al., 2013; Bekebrede et al., 2020). Moreover, the application of targeted mutagenesis techniques to *Rickettsia* and *Ehrlichia* species is on the horizon (McClure et al., 2017). Combined with advanced analysis of functional genes in ticks along with molecular and cellular techniques to manipulate ticks, it is expected that *Ehrlichia* Himer1 transposon insertional mutant libraries will facilitate this line of investigation. Further experimental discoveries of bacterial factors and their functions during the natural life cycle of *Ehrlichia*—in which humans are merely accidental hosts—are expected to reveal the remarkable molecular evolution of these tick-borne pathogens and inform the development of effective therapeutic strategies and preventative measures for diseases caused by *Ehrlichia* species.

## Author Contributions

The author confirms being the sole contributor of this review and has approved it for publication.

## Conflict of Interest

The author declares that the research was conducted in the absence of any commercial or financial relationships that could be construed as a potential conflict of interest.

## Publisher’s Note

All claims expressed in this article are solely those of the authors and do not necessarily represent those of their affiliated organizations, or those of the publisher, the editors and the reviewers. Any product that may be evaluated in this article, or claim that may be made by its manufacturer, is not guaranteed or endorsed by the publisher.

## References

[B1] Adams D. A. Thomas K. R. Jajosky R. A. Foster L. Baroi G. Sharp P.. (2017). Summary of Notifiable Infectious Diseases and Conditions - United States, 2015. MMWR Morb Mortal Wkly Rep. 64, 1–143. doi: 10.15585/mmwr.mm6453a1 28796757

[B2] Alkishe A. Raghavan R. K. Peterson A. T. (2021). Likely Geographic Distributional Shifts Among Medically Important Tick Species and Tick-Associated Diseases Under Climate Change in North America: A Review. Insects 12, 1–25. doi: 10.3390/insects12030225 PMC8001278 33807736

[B3] Alrutz M. A. Srivastava A. Wong K. W. D’Souza-Schorey C. Tang M. Ch’Ng L. E.. (2001). Efficient Uptake of Yersinia Pseudotuberculosis *via* Integrin Receptors Involves a Rac1-Arp 2/3 Pathway That Bypasses N-WASP Function. Mol. Microbiol. 42, 689–703. doi: 10.1046/j.1365-2958.2001.02676.x 11722735

[B4] Alvarez-Martinez C. E. Christie P. J. (2009). Biological Diversity of Prokaryotic Type IV Secretion Systems. Microbiol. Mol. Biol. Rev. 73, 775–808. doi: 10.1128/MMBR.00023-09 19946141 PMC2786583

[B5] Barnewall R. E. Ohashi N. Rikihisa Y. (1999). *Ehrlichia chaffeensis* and *E. sennetsu*, But Not the Human Granulocytic Ehrlichiosis Agent, Colocalize With Transferrin Receptor and Up-Regulate Transferrin Receptor mRNA by Activating Iron-Responsive Protein 1. Infect. Immun. 67, 2258–2265. doi: 10.1128/IAI.67.5.2258-2265.1999 10225882 PMC115965

[B6] Barnewall R. E. Rikihisa Y. (1994). Abrogation of Gamma Interferon-Induced Inhibition of *Ehrlichia Chaffeensis* Infection in Human Monocytes With Iron-Transferrin. Infect. Immun. 62, 4804–4810. doi: 10.1128/iai.62.11.4804-4810.1994 7927758 PMC303190

[B7] Barnewall R. E. Rikihisa Y. Lee E. H. (1997). *Ehrlichia chaffeensis* Inclusions Are Early Endosomes Which Selectively Accumulate Transferrin Receptor. Infect. Immun. 65, 1455–1461. doi: 10.1128/iai.65.4.1455-1461.1997 9119487 PMC175153

[B8] Bekebrede H. Lin M. Teymournejad O. Rikihisa Y. (2020). Discovery of *In Vivo* Virulence Genes of Obligatory Intracellular Bacteria by Random Mutagenesis. Front. Cell Infect. Microbiol. 10, 2. doi: 10.3389/fcimb.2020.00002 32117791 PMC7010607

[B9] Biggs H. M. Behravesh C. B. Bradley K. K. Dahlgren F. S. Drexler N. A. Dumler J. S.. (2016). Diagnosis and Management of Tickborne Rickettsial Diseases: Rocky Mountain Spotted Fever and Other Spotted Fever Group Rickettsioses, Ehrlichioses, and Anaplasmosis - United States. MMWR Recomm Rep. 65, 1–44. doi: 10.15585/mmwr.rr6502a1 27172113

[B10] Bokoch G. M. Bohl B. P. Chuang T. H. (1994). Guanine Nucleotide Exchange Regulates Membrane Translocation of Rac/Rho GTP-Binding Proteins. J. Biol. Chem. 269, 31674–31679. doi: 10.1016/S0021-9258(18)31748-4 7989340

[B11] Bokoch G. M. Zhao T. (2006). Regulation of the Phagocyte NADPH Oxidase by Rac GTPase. Antioxid Redox Signal 8, 1533–1548. doi: 10.1089/ars.2006.8.1533 16987009

[B12] Bosse T. Ehinger J. Czuchra A. Benesch S. Steffen A. Wu X.. (2007). Cdc42 and Phosphoinositide 3-Kinase Drive Rac-Mediated Actin Polymerization Downstream of C-Met in Distinct and Common Pathways. Mol. Cell Biol. 27, 6615–6628. doi: 10.1128/MCB.00367-07 17682062 PMC2099217

[B13] Budachetri K. Teymournejad O. Lin M. Yan Q. Mestres-Villanueva M. Brock G. N.. (2020). An Entry-Triggering Protein of Ehrlichia Is a New Vaccine Candidate Against Tick-Borne Human Monocytic Ehrlichiosis. mBio 11, 1–13. doi: 10.1128/mBio.00895-20 PMC7387794 32723916

[B14] Byerly C. D. Patterson L. L. McBride J. W. (2021). Ehrlichia TRP Effectors: Moonlighting, Mimicry and Infection. Pathog. Dis. 79, 1–16. doi: 10.1093/femspd/ftab026 PMC8112483 33974702

[B15] Carabeo R. A. Grieshaber S. S. Hasenkrug A. Dooley C. Hackstadt T. (2004). Requirement for the Rac GTPase in Chlamydia Trachomatis Invasion of Non-Phagocytic Cells. Traffic 5, 418–425. doi: 10.1111/j.1398-9219.2004.00184.x 15117316

[B16] Cheng C. Nair A. D. Indukuri V. V. Gong S. Felsheim R. F. Jaworski D.. (2013). Targeted and Random Mutagenesis of *Ehrlichia Chaffeensis* for the Identification of Genes Required for *In Vivo* Infection. PloS Pathog. 9, e1003171. doi: 10.1371/journal.ppat.1003171 23459099 PMC3573109

[B17] Dawson J. E. Anderson B. E. Fishbein D. B. Sanchez J. L. Goldsmith C. S. Wilson K. H.. (1991). Isolation and Characterization of an *Ehrlichia* sp. From a Patient Diagnosed With Human Ehrlichiosis. J. Clin. Microbiol. 29, 2741–2745. doi: 10.1128/jcm.29.12.2741-2745.1991 1757543 PMC270425

[B18] Debeurme F. Picciocchi A. Dagher M. C. Grunwald D. Beaumel S. Fieschi F.. (2010). Regulation of NADPH Oxidase Activity in Phagocytes: Relationship Between FAD/NADPH Binding and Oxidase Complex Assembly. J. Biol. Chem. 285, 33197–33208. doi: 10.1074/jbc.M110.151555 20724480 PMC2963400

[B19] Deretic V. (2012). Autophagy: An Emerging Immunological Paradigm. J. Immunol. 189, 15–20. doi: 10.4049/jimmunol.1102108 22723639 PMC3382968

[B20] Dunning Hotopp J. C. Lin M. Madupu R. Crabtree J. Angiuoli S. V. Eisen J.. (2006). Comparative Genomics of Emerging Human Ehrlichiosis Agents. PloS Genet. 2, e21. doi: 10.1371/journal.pgen.0020021 16482227 PMC1366493

[B21] Gabig T. G. Babior B. M. (1981). The Killing of Pathogens by Phagocytes. Annu. Rev. Med. 32, 313–326. doi: 10.1146/annurev.me.32.020181.001525 7013670

[B22] Geiszt M. Dagher M. C. Molnar G. Havasi A. Faure J. Paclet M. H.. (2001). Characterization of Membrane-Localized and Cytosolic Rac-GTPase-Activating Proteins in Human Neutrophil Granulocytes: Contribution to the Regulation of NADPH Oxidase. Biochem. J. 355, 851–858. doi: 10.1042/bj3550851 11311150 PMC1221803

[B23] Gillespie J. J. Brayton K. A. Williams K. P. Diaz M. A. Brown W. C. Azad A. F.. (2010). Phylogenomics Reveals a Diverse Rickettsiales Type IV Secretion System. Infect. Immun. 78, 1809–1823. doi: 10.1128/IAI.01384-09 20176788 PMC2863512

[B24] Holley A. K. Dhar S. K. Xu Y. St Clair D. K. (2012). Manganese Superoxide Dismutase: Beyond Life and Death. Amino Acids 42, 139–158. doi: 10.1007/s00726-010-0600-9 20454814 PMC2975048

[B25] Humphreys D. Davidson A. C. Hume P. J. Makin L. E. Koronakis V. (2013). Arf6 Coordinates Actin Assembly Through the WAVE Complex, a Mechanism Usurped by Salmonella to Invade Host Cells. Proc. Natl. Acad. Sci. U. S. A. 110, 16880–16885. doi: 10.1073/pnas.1311680110 24085844 PMC3801044

[B26] Inoue A. Sawata S. Y. Taira K. Wadhwa R. (2007). Loss-Of-Function Screening by Randomized Intracellular Antibodies: Identification of hnRNP-K as a Potential Target for Metastasis. Proc. Natl. Acad. Sci. U. S. A. 104, 8983–8988. doi: 10.1073/pnas.0607595104 17483488 PMC1885614

[B27] Levine B. Mizushima N. Virgin H. W. (2011). Autophagy in Immunity and Inflammation. Nature 469, 323–335. doi: 10.1038/nature09782 21248839 PMC3131688

[B28] Lin M. Grandinetti G. Hartnell L. M. Bliss D. Subramaniam S. Rikihisa Y. (2020). Host Membrane Lipids Are Trafficked to Membranes of Intravacuolar Bacterium Ehrlichia Chaffeensis. Proc. Natl. Acad. Sci. U. S. A. 117, 8032–8043. doi: 10.1073/pnas.1921619117 32193339 PMC7149431

[B29] Lin M. Liu H. Xiong Q. Niu H. Cheng Z. Yamamoto A.. (2016). Ehrlichia Secretes Etf-1 to Induce Autophagy and Capture Nutrients for Its Growth Through RAB5 and Class III Phosphatidylinositol 3-Kinase. Autophagy 12, 2145–2166. doi: 10.1080/15548627.2016.1217369 27541856 PMC5103349

[B30] Lin M. Rikihisa Y. (2003). *Ehrlichia chaffeensis* and *Anaplasma phagocytophilum* Lack Genes for Lipid A Biosynthesis and Incorporate Cholesterol for Their Survival. Infect. Immun. 71, 5324–5331. doi: 10.1128/IAI.71.9.5324-5331.2003 12933880 PMC187327

[B31] Lin M. Rikihisa Y. (2007). Degradation of P22phox and Inhibition of Superoxide Generation by *Ehrlichia chaffeensis* in Human Monocytes. Cell Microbiol. 9, 861–874. doi: 10.1111/j.1462-5822.2006.00835.x 17087735

[B32] Liu H. Bao W. Lin M. Niu H. Rikihisa Y. (2012). Ehrlichia Type IV Secretion Effector ECH0825 Is Translocated to Mitochondria and Curbs ROS and Apoptosis by Upregulating Host MnSOD. Cell Microbiol. 14, 1037–1050. doi: 10.1111/j.1462-5822.2012.01775.x 22348527 PMC3371182

[B33] Liu Y. Zhang Z. Jiang Y. Zhang L. Popov V. L. Zhang J.. (2011). Obligate Intracellular Bacterium *Ehrlichia* Inhibiting Mitochondrial Activity. Microbes Infect. 13, 232–238. doi: 10.1016/j.micinf.2010.10.021 21070861 PMC3031727

[B34] Madison-Antenucci S. Kramer L. D. Gebhardt L. L. Kauffman E. (2020). Emerging Tick-Borne Diseases. Clin. Microbiol. Rev. 33, 1–18. doi: 10.1128/CMR.00083-18 PMC6941843 31896541

[B35] Maeda K. Markowitz N. Hawley R. C. Ristic M. Cox D. McDade J. E. (1987). Human Infection With *Ehrlichia canis*, A Leukocytic Rickettsia. N. Engl. J. Med. 316, 853–856. doi: 10.1056/NEJM198704023161406 3029590

[B36] McBride J. W. Walker D. H. (2011). Molecular and Cellular Pathobiology of Ehrlichia Infection: Targets for New Therapeutics and Immunomodulation Strategies. Expert Rev. Mol. Med. 13, e3. doi: 10.1017/S1462399410001730 21276277 PMC3767467

[B37] McClure E. E. Chavez A. S. O. Shaw D. K. Carlyon J. A. Ganta R. R. Noh S. M.. (2017). Engineering of Obligate Intracellular Bacteria: Progress, Challenges and Paradigms. Nat. Rev. Microbiol. 15, 544–558. doi: 10.1038/nrmicro.2017.59 28626230 PMC5557331

[B38] Mohan Kumar D. Lin M. Xiong Q. Webber M. J. Kural C. Rikihisa Y. (2015). EtpE Binding to DNase X Induces Ehrlichial Entry *via* CD147 and hnRNP-K Recruitment, Followed by Mobilization of N-WASP and Actin. MBio 6, 1–15. doi: 10.1128/mBio.01541-15 PMC4631803 26530384

[B39] Mohan Kumar D. Yamaguchi M. Miura K. Lin M. Los M. Coy J. F.. (2013). *Ehrlichia chaffeensis* Uses Its Surface Protein EtpE to Bind GPI-Anchored Protein DNase X and Trigger Entry Into Mammalian Cells. PloS Pathog. 9, e1003666. doi: 10.1371/journal.ppat.1003666 24098122 PMC3789761

[B40] Mott J. Barnewall R. E. Rikihisa Y. (1999). Human Granulocytic Ehrlichiosis Agent and *Ehrlichia chaffeensis* Reside in Different Cytoplasmic Compartments in HL-60 Cells. Infect. Immun. 67, 1368–1378. doi: 10.1128/IAI.67.3.1368-1378.1999 10024584 PMC96470

[B41] Niu H. Kozjak-Pavlovic V. Rudel T. Rikihisa Y. (2010). *Anaplasma phagocytophilum* Ats-1 Is Imported Into Host Cell Mitochondria and Interferes With Apoptosis Induction. PloS Pathog. 6, e1000774. doi: 10.1371/journal.ppat.1000774 20174550 PMC2824752

[B42] Paddock C. D. Childs J. E. (2003). *Ehrlichia chaffeensis*: A Prototypical Emerging Pathogen. Clin. Microbiol. Rev. 16, 37–64. doi: 10.1128/CMR.16.1.37-64.2003 12525424 PMC145301

[B43] Panday A. Sahoo M. K. Osorio D. Batra S. (2015). NADPH Oxidases: An Overview From Structure to Innate Immunity-Associated Pathologies. Cell Mol. Immunol. 12, 5–23. doi: 10.1038/cmi.2014.89 25263488 PMC4654378

[B44] Pfanner N. Truscott K. N. (2002). Powering Mitochondrial Protein Import. Nat. Struct. Biol. 9, 234–236. doi: 10.1038/nsb0402-234 11914726

[B45] Raspe M. Gillis J. Krol H. Krom S. Bosch K. van Veen H.. (2009). Mimicking Proteasomal Release of Polyglutamine Peptides Initiates Aggregation and Toxicity. J. Cell Sci. 122, 3262–3271. doi: 10.1242/jcs.045567 19690053

[B46] Ravikumar B. Imarisio S. Sarkar S. O’Kane C. J. Rubinsztein D. C. (2008). Rab5 Modulates Aggregation and Toxicity of Mutant Huntingtin Through Macroautophagy in Cell and Fly Models of Huntington Disease. J. Cell Sci. 121, 1649–1660. doi: 10.1242/jcs.025726 18430781 PMC2635563

[B47] Rikihisa Y. (1991). The Tribe *Ehrlichieae* and Ehrlichial Diseases. Clin. Microbiol. Rev. 4, 286–308. doi: 10.1128/CMR.4.3.286 1889044 PMC358200

[B48] Rikihisa Y. (2010). *Anaplasma phagocytophilum* and *Ehrlichia chaffeensis*: Subversive Manipulators of Host Cells. Nat. Rev. Microbiol. 8, 328–339. doi: 10.1038/nrmicro2318 20372158

[B49] Rikihisa Y. (2011). Mechanisms of Obligatory Intracellular Infection With Anaplasma Phagocytophilum. Clin. Microbiol. Rev. 24, 469–489. doi: 10.1128/CMR.00064-10 21734244 PMC3131063

[B50] Rikihisa Y. (2015). Molecular Pathogenesis of *Ehrlichia chaffeensis* Infection. Annu. Rev. Microbiol. 69, 283–304. doi: 10.1146/annurev-micro-091014-104411 26488275

[B51] Rikihisa Y. (2017). Role and Function of the Type IV Secretion System in *Anaplasma* and *Ehrlichia* Species. Curr. Top. Microbiol. Immunol. 413, 297–321. doi: 10.1007/978-3-319-75241-9_12 29536364

[B52] Rikihisa Y. (2019). Subversion of RAB5-Regulated Autophagy by the Intracellular Pathogen Ehrlichia chaffeensis. Small GTPases 10, 343–349. doi: 10.1080/21541248.2017.1332506 28650718 PMC6748376

[B53] Roberts A. W. Kim C. Zhen L. Lowe J. B. Kapur R. Petryniak B.. (1999). Deficiency of the Hematopoietic Cell-Specific Rho Family GTPase Rac2 Is Characterized by Abnormalities in Neutrophil Function and Host Defense. Immunity 10, 183–196. doi: 10.1016/S1074-7613(00)80019-9 10072071

[B54] Seifert R. Rosenthal W. Schultz G. (1986). Guanine Nucleotides Stimulate NADPH Oxidase in Membranes of Human Neutrophils. FEBS Lett. 205, 161–165. doi: 10.1016/0014-5793(86)80886-9 3017756

[B55] Sharma P. Teymournejad O. Rikihisa Y. (2017). Peptide Nucleic Acid Knockdown and Intra-Host Cell Complementation of Ehrlichia Type IV Secretion System Effector. Front. Cell Infect. Microbiol. 7, 228. doi: 10.3389/fcimb.2017.00228 28638803 PMC5461285

[B56] Teymournejad O. Lin M. Rikihisa Y. (2017). Ehrlichia Chaffeensis and Its Invasin EtpE Block Reactive Oxygen Species Generation by Macrophages in a DNase X-Dependent Manner. MBio 8. doi: 10.1128/mBio.01551-17 PMC5698551 29162709

[B57] Teymournejad O. Rikihisa Y. (2020). Ehrlichia chaffeensis Uses an Invasin To Suppress Reactive Oxygen Species Generation by Macrophages *via* CD147-Dependent Inhibition of Vav1 To Block Rac1 Activation. mBio 11, 1–14. doi: 10.1128/mBio.00267-20 PMC7175088 32317318

[B58] Wurm C. A. Neumann D. Lauterbach M. A. Harke B. Egner A. Hell S. W.. (2011). Nanoscale Distribution of Mitochondrial Import Receptor Tom20 Is Adjusted to Cellular Conditions and Exhibits an Inner-Cellular Gradient. Proc. Natl. Acad. Sci. U. S. A. 108, 13546–13551. doi: 10.1073/pnas.1107553108 21799113 PMC3158204

[B59] Yan Q. Lin M. Huang W. Teymournejad O. Johnson J. M. Hays F. A.. (2018). Ehrlichia Type IV Secretion System Effector Etf-2 Binds to Active RAB5 and Delays Endosome Maturation. Proc. Natl. Acad. Sci. U. S. A. 115, E8977–E8986. doi: 10.1073/pnas.1806904115 30181274 PMC6156607

[B60] Yan Q. Zhang W. Lin M. Teymournejad O. Budachetri K. Lakritz J.. (2021). Iron Robbery by Intracellular Pathogen *via* Bacterial Effector-Induced Ferritinophagy. Proc. Natl. Acad. Sci. U. S. A. 118, e2026598118. doi: 10.1073/pnas.2026598118 34074773 PMC8201858

[B61] Yoo Y. Wu X. Egile C. Li R. Guan J. L. (2006). Interaction of N-WASP With hnRNPK and Its Role in Filopodia Formation and Cell Spreading. J. Biol. Chem. 281, 15352–15360. doi: 10.1074/jbc.M511825200 16574661

[B62] Zhang W. Lin M. Yan Q. Budachetri K. Hou L. Sahni A.. (2021). An Intracellular Nanobody Targeting T4SS Effector Inhibits Ehrlichia Infection. Proc. Natl. Acad. Sci. U. S. A. 118, e2024102118. doi: 10.1073/pnas.2024102118 33903242 PMC8106314

[B63] Zhao T. Benard V. Bohl B. P. Bokoch G. M. (2003). The Molecular Basis for Adhesion-Mediated Suppression of Reactive Oxygen Species Generation by Human Neutrophils. J. Clin. Invest. 112, 1732–1740. doi: 10.1172/JCI19108 14660749 PMC281647

[B64] Zhao X. Carnevale K. A. Cathcart M. K. (2003). Human Monocytes Use Rac1, Not Rac2, in the NADPH Oxidase Complex. J. Biol. Chem. 278, 40788–40792. doi: 10.1074/jbc.M302208200 12912997

